# Automated detection of the CT-angiography spot sign in acute intracerebral hemorrhage

**DOI:** 10.3389/fradi.2026.1898570

**Published:** 2026-10-26

**Authors:** Dar Dowlatshahi, Santanu Chakraborty, Tim Ramsay, Matthew J. Hogan

**Affiliations:** 1 Department of Medicine (Neurology), University of Ottawa, Ottawa, ON, Canada; 2 Neurosciences Program, Ottawa Hospital Research Institute, Ottawa, ON, Canada; 3 Department of Medical Imaging (Neuroradiology), University of Ottawa, Ottawa, ON, Canada; 4 Methodological and Implementation Research Program, Ottawa Hospital Research Institute, Ottawa, ON, Canada

**Keywords:** automation, contrast extravasation, CT angiography, intracerebral hemorrhage, spot sign

## Abstract

Early hematoma expansion is a major determinant of outcome after intracerebral hemorrhage (ICH). The CTA spot sign predicts hematoma expansion and poor outcome but requires specialized expertise for interpretation. Automated detection facilitates imaging-based selection strategies for hemostatic therapies. We evaluated the diagnostic performance of an automated algorithm for spot sign detection using multiphase CTA. We analyzed patients presenting with spontaneous ICH within 4.5 h of symptom onset who underwent multiphase CTA. Fifty scans were randomly selected from a prospective registry. The automated algorithm independently classified each scan as spot sign positive or negative. An expert neuroradiologist served as reference standard. Sensitivity for spot sign detection was 95.7%, specificity was 74.1%. In this pilot study, automated detection of the CTA spot sign on dynamic CTA was feasible and demonstrated high sensitivity but moderate specificity, supporting further independent validation of CARL. Such tools may standardize spot sign identification, support clinical trial enrollment, and have the potential to facilitate rapid selection of patients at high risk of hematoma expansion.

## Introduction

Early hematoma expansion is a major determinant of clinical outcome and mortality after intracerebral hemorrhage (ICH) and represents an important therapeutic target (1). The computed tomography angiography (CTA) spot sign has emerged as a robust imaging biomarker that predicts hematoma expansion, poor functional outcome, and mortality (2). When identified early after symptom onset, the spot sign likely represents contrast extravasation and ongoing bleeding, thereby identifying patients at highest risk of clinical deterioration.

The recently published FASTEST trial evaluated recombinant activated factor VII (rFVIIa) for the treatment of hematoma expansion in patients presenting within 2 h of ICH onset (3). Although the overall trial did not demonstrate a statistically significant improvement in functional outcomes, prespecified analyses demonstrated a strong signal suggesting that rFVIIa reduced hematoma expansion and improved clinical outcomes among patients with CTA spot sign–positive ICH. These findings highlight the importance of early identification of patients at high risk of hematoma expansion and suggest that imaging-based selection strategies may enhance the effectiveness of hemostatic therapies.

Despite its clinical utility, interpretation of the CTA spot sign requires specialized expertise and training, which may limit its widespread adoption in acute stroke workflows. Automated imaging platforms are increasingly used to streamline acute stroke imaging interpretation, including automated detection of ICH and hematoma volume quantification. Accordingly, we developed a fully automated software, Computer Assisted Recognition of Leakage (CARL), designed to detect the CTA spot sign using multiphase CTA data. In this study, we aimed to evaluate the diagnostic performance of CARL for spot sign detection compared with expert neuroradiologist interpretation as the reference standard.

## Methods

### Participants

We analyzed patients from a prospectively collected imaging registry of consecutive adults presenting with spontaneous ICH at The Ottawa Hospital, a tertiary academic center in Ottawa, Canada (4). The Ottawa Health Science Network Research Ethics Board approved the registry, which included patients aged ≥18 years who present within 4.5 h of symptom onset and underwent multiphase CTA as part of a standardized acute stroke imaging protocol between 2013 and 2018. We excluded patients with known secondary causes of hemorrhage, including trauma, intracranial malignancy, aneurysm, or arteriovenous malformation.

### Imaging

We performed whole-brain dynamic CT using a 320-row volume CT scanner (Aquilion ONE™, Toshiba, Japan), as previously described (4). Briefly, we acquired 19 whole-brain image volumes over 53 s following intravenous infusion of iodinated contrast. We used interscan intervals of 2 s during peak contrast bolus passage and 5-second intervals thereafter. Image voxel dimensions were 0.43 × 0.43 × 0.5 mm.

### CARL technology

CARL is an automated algorithm developed within the MATLAB (MathWorks, Inc) environment to identify contrast leakage on multiphase CTA data. The software organizes the image series into a four-dimensional dataset, representing three-dimensional spatial information across sequential time points. An intracranial tissue mask is generated to exclude extracranial structures, and temporal variation in voxel attenuation is used to identify candidate voxels demonstrating changes in contrast signal over time. Voxels without sufficient temporal signal variation are excluded from further analysis. CARL groups spatially contiguous voxels showing contrast signal into clusters and tracks their evolution at sequential time points. As voxels adjacent to a cluster acquire contrast signal they are then joined to the respective cluster. Time–density curves derived from vascular structures are used as reference arterial and venous enhancement patterns, allowing clusters with corresponding vascular enhancement characteristics to be identified and removed. Among the remaining clusters, CARL identifies candidate bleeding sites based on progressive spatial expansion over time (Figure 1), consistent with contrast extravasation.

**Figure 1:**
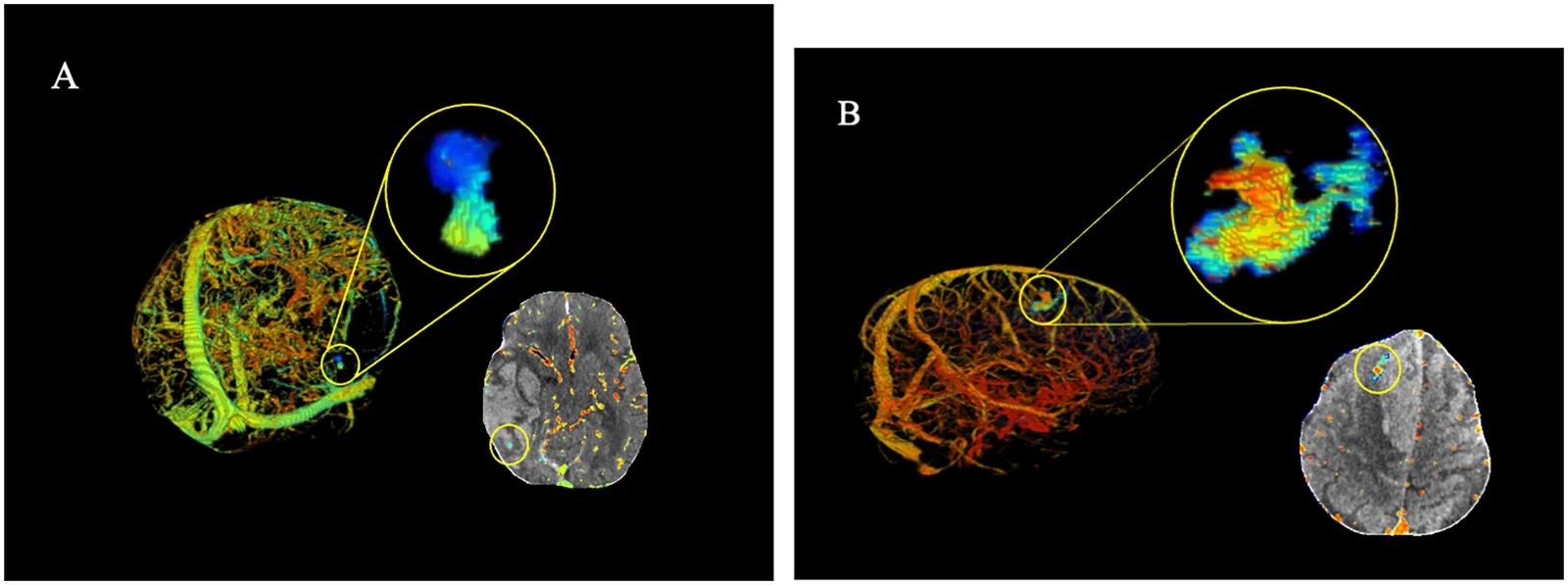
Spatiotemporal characterization of the CTA spot sign using voxel-wise contrast arrival time mapping. The images in panels **A** and **B** demonstrate three-dimensional renderings of dynamic CT angiography on the left, including the spot sign within an intracerebral hemorrhage (yellow circle). Each voxel within the rendering is colour-coded according to contrast signal start time, with early-phase enhancement shown in red and progressively later enhancement displayed along the color spectrum toward blue. The insets within each panel provide a magnified view of the spot sign, illustrating the temporal gradient of contrast appearance and spatial expansion over time. This spectrum of contrast start times defines the spot sign as a dynamic process rather than a static focus of enhancement. This progressing spatial expansion of contrast enhancement along with the temporal profile of contrast concentration is used to identify and visualize the spot sign within the dynamic CT angiogram.

### Statistical analysis

This was a pilot diagnostic accuracy study. After consultation with the Ottawa Methods Centre (https://ohri.ca/en/who-we-are/core-facilities-and-platforms/ottawa-methods-centre), a pragmatic sample of 50 dynamic CTA scans was randomly selected from the parent registry while preserving the observed spot-sign prevalence (45%) (4), in order to obtain preliminary estimates of sensitivity and specificity of the automated algorithm against expert reference interpretation. CARL is a deterministic rule-based algorithm rather than a trained machine-learning model and was locked before evaluation of this cohort; although the evaluation cases were selected separately, complete historical non-overlap with cases used during earlier iterative algorithm development cannot be guaranteed. We selected 22 spot sign–positive and 28 spot sign–negative cases. CARL independently classified each scan as spot sign positive or negative. We compared these classifications against a reference standard interpretation performed by a board-certified expert neuroradiologist (SC) with more than 15 years of experience in spot sign assessment and who originally described the dynamic CTA spot sign (5, 6). The expert reviewed imaging using a dedicated clinically-approved PACS terminal blind to patient information and followup scans. The initial expert interpretation was completed before CARL was applied and was fully blinded to CARL output; after discrepant cases were identified, an unblinded *post hoc* consensus review was undertaken specifically to determine whether CARL had identified a true spot sign missed on initial expert review. Both CARL and expert were blind to clinical and demographic information. We calculated the sensitivity and specificity of CARL for spot sign detection using the expert interpretation as the gold standard and derived 95% confidence intervals using exact binomial (Clopper–Pearson) methods.

## Results

Fifty dynamic CTA studies were analyzed, including 22 spot sign–positive and 28 spot sign–negative cases based on initial radiologist interpretation (Table 1). Using the neuroradiologist assessment as the expert reference standard, CARL yielded 21 true positives, 20 true negatives, 8 false positives, and 1 false negative; nine cases showed disagreement between CARL and the expert interpretation. All discrepant cases underwent joint review by the neuroradiologist and two experienced stroke neurologists with extensive experience with the CTA spot sign (DD and MH) to establish a consensus classification. In one instance initially classified as spot sign–negative by the neuroradiologist but positive by CARL, consensus review led to unanimous reclassification as spot sign–positive, thereby yielding a reclassified 22 true positives, 20 true negatives, 7 false positives, and 1 false negative. The remaining discrepant cases reflected instances in which CARL identified potential leakage that was not confirmed on expert review, and one missed spot in a scan with movement artifact. Against the initial independent expert reference standard, CARL had a sensitivity of 95.5% (95% CI 77.2–99.9) and specificity of 71.4% (95% CI 51.3–86.8); after *post hoc* consensus reclassification of one discrepant case, sensitivity was 95.7% (95% CI 78.1–99.9) and specificity 74.1% (95% CI 53.7–88.9).

**Table 1:** Baseline demographic and imaging characteristic of study participants.

Age, median (IQR)	72 (63.5, 81.8)
Female sex	28/50 (56%)
Spot positive (initial classification)	22/50 (44%)
Spot positive (reclassification)	23/50 (46%)
Hematoma volume (parenchymal), median (IQR)	21 mL (6.6, 55.2)
Total hematoma volume (parenchymal + ventricular), median (IQR)	24.9 mL (7.2, 57.9)
Intraventricular hemorrhage (%)	14/50 (28%)
Onset to imaging time, median (IQR)	89 min (70, 133.8)

## Discussion

Early identification of active bleeding remains central for acute hemostatic therapies in ICH, as recent clinical trial data have renewed interest in imaging-based selection strategies (3, 7). In the FASTEST trial^7^, treatment with rFVIIa was associated with a substantially greater reduction in hematoma growth among patients with a CTA spot sign compared with those without one, with decreases of approximately 9.4 mL in ICH growth and 14.1 mL in combined ICH plus intraventricular hemorrhage growth, whereas only minimal reductions were observed in patients without a spot sign. These findings support the concept that imaging biomarkers of active bleeding identify a subgroup in whom hemostatic therapy may have the greatest biological effect, reinforcing its role as a key imaging biomarker for treatment selection. As therapies targeting ultra-early hematoma expansion continue to emerge, rapid and reliable identification of spot-positive patients will become increasingly important. In this context, tools that enable rapid and consistent identification of the CTA spot sign, irrespective of local radiological expertise, could play an important role in both clinical decision-making and trial enrollment. In this study, we report that an automated algorithm detected the spot sign with high sensitivity and moderate specificity compared with expert interpretation. These findings suggest that automated detection of spot sign is feasible.

Automated imaging platforms are increasingly integrated into acute stroke workflows. Tools such as RapidAI played a key role in enabling late-window thrombectomy trials (8, 9) and were subsequently adopted into routine clinical practice across many comprehensive stroke centers. Similarly, platforms such as Viz.ai and RapidAI have supported intracerebral hemorrhage trial enrollment by providing rapid automated volumetric assessments for screening and eligibility. Our findings extend this paradigm by demonstrating the feasibility of automatically detecting an imaging biomarker that may aid prognostication, facilitate enrollment into ongoing clinical trials, and potentially support selection of patients for hemostatic therapy. The present study evaluated automated detection of the CTA spot sign but did not directly assess whether CARL classification predicts subsequent hematoma expansion, clinical outcome, or response to hemostatic therapy; these potential downstream applications require prospective validation.

This study has limitations. The sample size was modest and intentionally selected to preserve the spot sign prevalence observed in the parent cohort, which may limit generalizability. External validation in independent cohorts is needed. Moreover, the moderate specificity suggests that the current prototype may be sensitive to subtle signal changes, highlighting the need for further refinement. In addition, CARL was evaluated only using 19-volume dynamic CTA acquired on a 320-row scanner, these findings establish proof of concept under this specific acquisition protocol and should not be generalized to conventional single-phase or limited multiphase CTA; validation on other scanners and lower-temporal-resolution protocols is required. Finally, the algorithm currently operates within a research-based computational environment and will require optimization and integration into vendor platforms for real-time clinical use.

These preliminary findings provide proof of concept for automated spot-sign detection, but larger independent studies are required to establish the diagnostic performance and generalizability of CARL. Such tools may help standardize detection across centers, support clinical trial enrollment, and facilitate rapid identification of patients most likely to benefit from hemostatic therapies.

## Data Availability

The raw data supporting the conclusions of this article will be made available by the authors, without undue reservation.
